# Paraganglioma of the endolarynx: a rare tumor in an uncommon location

**DOI:** 10.1186/1758-3284-2-2

**Published:** 2010-01-19

**Authors:** Joseph R Smolarz, Ehab Y Hanna, Michelle D Williams, Michael E Kupferman

**Affiliations:** 1 Department of Otorhinolaryngology, Head and Neck Surgery/Otolaryngology, The University of Texas Health Science Center at Houston, 6431 Fannin, Suite MSB 5.036, Houston, TX 77030, USA; 2 Department of Head and Neck Surgery, The University of Texas M.D. Anderson Cancer Center, 1400 Pressler, Unit 1445, Houston, TX 77030, USA; 3 Department of Pathology, The University of Texas M.D. Anderson Cancer Center, 1515 Holcombe Blvd, Unit 0085, Houston, TX 77030, USA

## Abstract

**Background:**

Less than 80 reported cases of paragangliomas of the larynx are reported in the literature. A role for external beam radiation in this disease has not yet been explored. We present four cases of laryngeal paragangliomas treated at a large tertiary-care cancer center over a 35-year period.

**Methods:**

124 cases of head and neck paragangliomas treated at a single institution from 1970 to 2005 were retrospectively studied. Patients with laryngeal paragangliomas were identified, and a comprehensive clinico-pathological review was undertaken.

**Results:**

We identified 4 patients with tumors arising in the larynx at the following subsites: supraglottis (2), glottis (1), and subglottis (1). Three patients were treated with surgery and one with definitive radiation alone.

**Conclusions:**

Laryngeal paragangliomas are rare tumors and are adequately treated with surgical resection. We also present one patient who was treated with radiation and had disease stabilization. Accurate histological classification is critical, and the role of genetic testing is emerging.

## Introduction

Paragangliomas of the laryngopharynx are rare tumors that are of neuroendocrine origin and arise from the neural crest-derived cells of the parasympathetic nervous system. Since its first description in the literature in 1955, fewer than 80 such cases have been reported [1]. These benign lesions have a clinical course marked by slow growth, with symptoms often mimicking those of a squamous cell carcinoma. Patients present with hoarseness or dysphasia, and a submucosal mass is evident on physical examination. The majority of these tumors arise in the supraglottic larynx, and 2% of these are malignant [1-3]. Since these tumors are from neural crest-derived cells, they usually appear adjacent to nerve structures, most commonly the superior laryngeal nerve or the recurrent laryngeal nerve[4]. While surgical resection is often curative, this approach is based upon small case series and retrospective data. Limited data is available on the role of external beam radiation therapy for the management of this disease. While radiation therapy has been utilized for the more common jugulo-tympanic paragangliomas, this modality has received little attention as a laryngeal preservation approach. In this study, we reviewed a single-institutional experience with laryngeal paragangliomas treated over a 35-year period.

## Methods - Study Design and Setting

All laryngeal paragangliomas treated at the University of Texas M.D. Anderson Cancer Center over the last 35 years were reviewed after IRB approval was obtained. We identified 124 patients with cervical paragangliomas, five of whom had tumors localized to the laryngopharynx. One patient presented to M.D. Anderson for a second opinion for a hypopharyngeal paraganglioma. This patient was eliminated from our study as he was lost to follow-up. Pathology reports, operative notes, video stroboscopic films and reports, radiologic imaging, and all clinical notes were reviewed for the remaining patients. A comprehensive review of the literature was also performed.

## Case Reports

### Case No. 1

A 67-year-old woman presented with voice changes. On flexible endoscopy, a submucosal mass was seen extending through the glottic aperture. The patient underwent an incisional biopsy, which revealed a paraganglioma by immunohistochemical and histopathological criteria. The patient was taken for surgery, and a direct laryngoscopy revealed a tumor originating in the subglottis, extending to the vocal folds at the anterior commissure. Due to the large pushing borders of the tumor that caused extensive damage to the thyroid and cricoid cartilage, a total laryngectomy was performed. Cartilage destruction with no apparent invasion of the larynx from the paraganglioma was evident on histologic evaluation. The patient remained free of disease and deceased from other causes two years later.

### Case No. 2

A-50 year-old man presented with a recurrent mass of the left supraglottic larynx that had been treated with a supraglottic laryngectomy eight months prior to presentation at our institution. A review of the pathology revealed a paraganglioma. He was treated with a total laryngectomy, but eight months after surgery, he presented with histologically-confirmed bilateral lung metastasis and brain metastasis. He was treated with systemic chemotherapy but ultimately developed spinal metastases and progressive pulmonary disease. The patient ultimately died from his disease 16 months after diagnosis.

### Case No. 3

**Figure 1:**
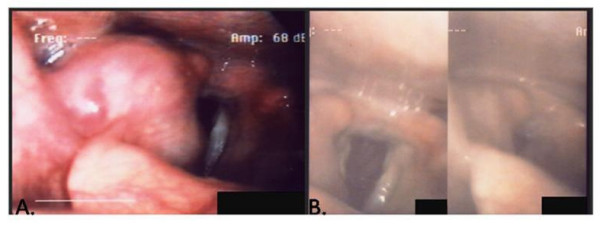
**Case No. 3 (A) Pretreatment stroboscopy revealed tumor involvement in the right AE fold, extending down into the right piriform sinus (B) Post-radiation - Only a small remnant of tumor remained after radiation and continued to be stable throughout follow-up**.

An 85-year-old woman presented to an outside institution with a two-year history of cough. A supraglottic lesion was noted on axial imaging of the neck, and a biopsy revealed a spindle cell neoplasm. This lesion was followed for one year, but the patient presented to our institution with progression of her disease, which included worsening of cough, new onset of hoarseness, and dysphasia with symptoms of aspiration. On flexible laryngoscopy, the patient had a right laryngeal mass emanating from the right false cord and extended to the aryepiglottic fold and epiglottis (Figure 1). Although surgery was recommended, the patient opted for external beam radiation. After five years of follow-up, the larynx lesion was still present as a pale 2 mm cystic area on the right aryepiglottic fold and had not grown in size since the radiation treatment.

### Case No. 4

**Figure 2:**
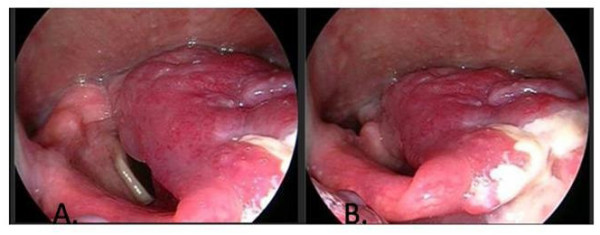
**Case No. 4 (A) A large laryngeal paraganglioma is noted in the left arytenoid area**. (B) There is involvement of the laryngeal and lingual surfaces of the epiglottis.

**Figure 3:**
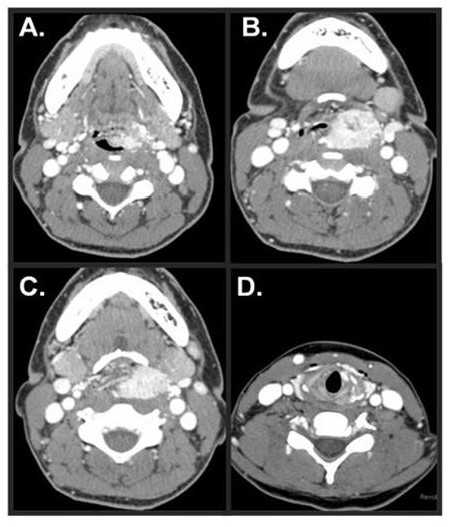
**Case No. 4 - (A) Epiglottic extension (B) Tumor pushing the airway to the right (C) Near-total laryngeal obstruction (D) Extension of tumor is found in the left anterior subglottis**.

**Figure 4:**
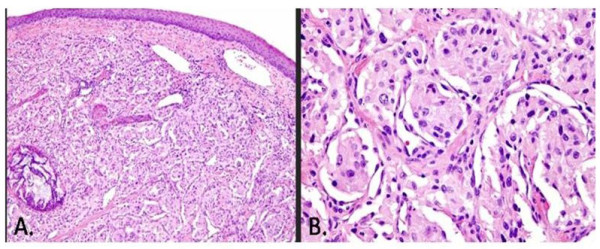
**Case No. 4 (A) Overlying squamous epithelium with the tumor in the submucosa**. The circular focus is a post-embolized vessel. (B) Higher power 400× - Zellballen nests of tumor cells with thin supporting sustenacular cells around each nest.

A 39-year-old black female was noted to have an asymptomatic glottic lesion at the time of a laparoscopic cholecystectomy for cholelithiasis. Intubation was performed without incident, though approximately one week later, she developed shortness of breath and airway compromise. A tracheostomy was placed at an outside facility. She subsequently underwent a panendoscopy and biopsy. On laryngoscopy, the lesion extended from the posterior aspect of the left arytenoid to along the aryepiglottic fold and false vocal cord onto the epiglottis (Figure 2). On CAT scan of the neck, axial imaging revealed an infiltrative and destructive mass of the left hemi-larynx. The enhancing tumor measured approximately 2.5 cm in AP dimension, 3 cm in width, and about 6 cm in height. The left subglottic component of the tumor probably extended for about 3-5 mm below the level of the true vocal cord (Figure 3). The patient subsequently underwent an extended supraglottic laryngectomy. Immunohistochemical stains for S-100, chromogranin, and synaptophysin were positive. Stains for pancytokeratin, EMA, and inhibin were negative. This immunohistochemical profile substantiated the diagnosis of paraganglioma (Figure 4). The patient was decannulated one month after surgery. On last follow-up 10 months after her operation, she had no evidence of disease. Furthermore, she had no difficulties with dysphasia or odynophagia, and the patient had functional speech with baseline hoarseness.

## Discussion

A report in 1994 by Ferlito et al critically evaluated all of the reported paragangliomas of the larynx and found that only 62 of these cases met their criteria for this diagnosis [1-3]. Since then, 14 more cases have been described in the surgical literature [5]. We add four more cases to the literature of laryngeal paragangliomas and report a single-institution experience with these rare lesions (Table 1).

**Table 1:** Summary of Laryngeal PG Patients

RESULTS	1	2	3	4
**Location**	**Subglottis**	**SG** **RM/DM**	**Right** **SG**	**Left SG/G** **Subglottis**
Sx vs Rad	Sx	Sx	Rad	Sx
Type of Sx	TL	SGL/TL	None	ESGL
Functioning	No	No	No	No
Multicentric	No	No	No	No
Misdiagnosis	No	Yes	Yes	No
Familial	No	No	No	No
Recurrence	No	Yes	No	No
Mortality	No	Yes	No	No
Metastasis	No	Yes	No	No

Sx- Surgery, Rad - Radiation, SGL - Supraglottic Laryngectomy, ESGL - Extended Supraglottic Laryngectomy, TL - Total Laryngectomy, SG - Supraglottis, G - Glottis, RM -Regional Metastasis, DM - Distant Metastasis

The management of laryngeal paraganglioma is usually surgical, and it often entails total laryngectomy. In selected cases, conservation surgical approaches have been suggested for oncologic control. Partial laryngectomy is a reasonable option in selected cases, particularly due to low likelihood of regional and distant metastasis [1,6]. Varied surgical techniques can be used utilized for laryngeal tumors, depending on the extent and site of the tumor. Transoral resections can provide oncological control for laryngeal tumors, though these approaches for paragangliomas have been discouraged in the literature due to difficult access in a major bleed [6]. Some authors advocate an external approach for excisional biopsy so the mucosa of the airway is not violated [3,6,7]. The anatomic considerations for partial laryngectomy are consistent with the operative approaches necessary for squamous cell histologies. However, due to the rarity of malignant paraganglioma of the larynx, a borderline tumor may be treated with a less extensive surgery, unlike squamous cell carcinoma. However, utilization of this treatment paradigm must be tempered by the skills of the surgeon and the reliability of the patient for intense post-operative surveillance.

A total of 23 inferior laryngeal paragangliomas have been reported in the literature, 11 of which presented in the subglottis. The other 12 presented as thyroid masses. Two patients with subglottic tumors required total laryngectomy, two required airway reconstruction, and one patient had recurrence [8]. These lesions that present in the subglottis have more dyspnea and shortness of breath and need careful consideration for airway management pre-operatively [7,9]. In our cohort, the patient with a subglottic paraganglioma required a total laryngectomy as the primary treatment. A conservation laryngeal approach was not attempted due to radiographic evidence of cartilage involvement without a clear plane for resection. Though recurrence rate is 4% in inferior laryngeal paraganglioma versus 17% reported for superior laryngeal paragangliomas, careful follow-up should be given to the inferior laryngeal cases due to the potential for rapid airway obstruction with recurrent or persistent disease [7,8].

Although paragangliomas are generally benign tumors [10], malignant paragangliomas have been reported with an incidence of 2% in some series [1-3]. However, pathologic findings cannot predict clinical behavior which requires metastases for designation as malignant. While the incidence of metastasis was initially reported to be as high as 25%, the critical review of Ferlito suggested that most of these tumors may indeed have been other histologies, including atypical carcinoid tumors, on further pathological review [1,3,5,11,12]. In Case No. 2, an excisional biopsy was preformed, and the initial diagnosis was malignant melanoma, nodular type, at an outside hospital. The pathological findings after the supraglottic laryngectomy were determined to be amelanotic melanoma as well. Even after the laryngectomy, the preliminary diagnosis was still the same. However, it was not until electron microscopy revealed paraganglioma of the larynx that the final diagnosis was determined. In Case No. 3, an outside pathology report revealed spindle cell tumor, but electron microscopy determined that the lesion was, in fact, paraganglioma.

Malignant paragangliomas more commonly metastasize distantly and rarely spread to the lateral neck lymph nodes. Thus, an elective neck dissection in a No neck with a biopsy confirmed malignant laryngeal paraganglioma is generally not advocated [1,2]. In our series, the single patient with cervical lymph node metastasis also developed distant metastases. Although a salvage total laryngectomy for local recurrence was performed, the patient ultimately succumbed to his disease.

As previously observed by multiple groups, neither radiation nor chemotherapy has been used successfully as an alternative to surgery in the management of laryngeal paragangliomas [2,5,13,14]. For the more common cervical paragangliomas, external beam radiation provides approximately 90% tumor control rate [15]. Our patient was monitored for five years and revealed reduction of the tumor mass without tumor progression after treatment with radiation.

The importance of genetic testing for mutations in the SHDB, SDHC, and SHDD genes has recently been highlighted [16]. Mutations in the *VHL *and *NF1 *genes are also associated with inherited paragangliomas. In particular, patients with SDHB mutations are at higher risk for developing malignant paragangliomas [17]. To date, there are only two cases of laryngeal paraganglioma that have been associated with multicentric head and neck paraganglioma syndrome. Though these particular patients did not undergo genetic testing, it is probable that gene mutations were the cause of multiple paragangliomas [18].

## Conclusion

Laryngopharyngeal paragangliomas are uncommon tumors that are conventionally treated with surgical resection for oncologic control. Close collaboration with an experienced pathologist is necessary to establish the diagnosis with immunohistochemical evaluation and electron microscopy to confirm the diagnosis. The evolving role of genetic testing is not defined, but it is likely that these four reported cases are sporadic. Surgical resection remains the standard of care for their treatment, although radiotherapy may be considered in selected patients. Close observation with serial imaging is necessary if this treatment approach is undertaken. Although malignant paragangliomas of the larynx have been reported, they are rare and metastasis should raise the question of misdiagnosis. Multi-institutional collaboration may provide greater insight into the true biological behavior of this disease and help optimize treatment approaches.

## Consent

Written informed consent was obtained from the patients for publication of this case report and accompanying images. A copy of the written consent is available for review by the Editor-in-Chief of this journal.

## Competing interests

The authors declare that they have no competing interests.

## Authors' contributions

JRS carried out the data collection and drafted the manuscript. EYH assisted in the concept of the design and editing the manuscript. MDW participated in the data collection. MEK conceived the study design, and helped in drafting and editing the manuscript. All authors read and approved the final manuscript.
